# A low-profile electromechanical packaging system for soft-to-flexible bioelectronic interfaces

**DOI:** 10.1063/5.0152509

**Published:** 2023-08-18

**Authors:** Florian Fallegger, Alix Trouillet, Florent-Valéry Coen, Giuseppe Schiavone, Stéphanie P. Lacour

**Affiliations:** Laboratory for Soft Bioelectronic Interfaces, Neuro-X Institute, Ecole Polytechnique Fédérale de Lausanne, Geneva, Switzerland

## Abstract

Interfacing the human body with the next generation of electronics requires technological advancement in designing and producing bioelectronic circuits. These circuits must integrate electrical functionality while simultaneously addressing limitations in mechanical compliance and dynamics, biocompatibility, and consistent, scalable manufacturing. The combination of mechanically disparate materials ranging from elastomers to inorganic crystalline semiconductors calls for modular designs with reliable and scalable electromechanical connectors. Here, we report on a novel interconnection solution for soft-to-flexible bioelectronic interfaces using a patterned and machined flexible printed circuit board, which we term FlexComb, interfaced with soft transducing systems. Using a simple assembly process, arrays of protruding “fingers” bearing individual electrical terminals are laser-machined on a standard flexible printed circuit board to create a comb-like structure, namely, the FlexComb. A matching pattern is also machined in the soft system to host and interlock electromechanically the FlexComb connections via a soft electrically conducting composite. We examine the electrical and electromechanical properties of the interconnection and demonstrate the versatility and scalability of the method through various customized submillimetric designs. In a pilot *in vivo* study, we validate the stability and compatibility of the FlexComb technology in a subdural electrocorticography system implanted for 6 months on the auditory cortex of a minipig. The FlexComb provides a reliable and simple technique to bond and connect soft transducing systems with flexible or rigid electronic boards, which should find many implementations in soft robotics and wearable and implantable bioelectronics.

## I. INTRODUCTION

Compliant (bio)electronic systems have emerged as a groundbreaking evolution in the field of bioengineering and medical devices.^1–4^ These miniaturized electronic circuits are designed to probe or modulate functions accommodating the body's inherent mechanical dynamics. With soft materials and stretchable designs, electronic interfaces can reliably conform and deform along with a mobile and non-planar host.^3,5,6^ Microfabrication focused on thin-films and thinned inorganic layers supports their integration within polymeric carriers, e.g., thermoplastics, such as polyimide^7^ or Parylene-C,^8^ and elastomers, such as polydimethylsiloxane (PDMS)^9^ or hydrogels.^6^ As the number of transducing channels and the need for wireless and on-board signal processing increase, hybrid systems hosting standard (rigid) with flexible and stretchable electronic modules have emerged as necessary platform solutions.^10–12^ They combine the electrical performance of rigid-material systems with the mechanical compliance of soft-material systems, but require robust, miniaturized, and versatile electromechanical connections to link heterogeneous modules in an integrated system.^13^ This electromechanical transition is a crucial component enabling the reliable function of hybrid (bio)electronic systems. When used in implantable scenarios, connection points must also sustain the harsh biochemical environment without releasing adverse by-products.

Based on a review of the current literature and our experience in implantable bioelectronic systems, the technology to connect soft and flexible or rigid modules should satisfy the following criteria: (1) compatibility with the manufacturing processes of both the soft and rigid modules, (2) follow a low geometrical profile neither to thicken nor widen the system at the point of connection, (3) scalability to connect tens to hundreds of signal channels, (4) negotiate the mechanical transition from the soft to rigid modules, (5) offer low electrical resistance independently of mechanical loading, and (6) ensure reliable and sufficient channel isolation. A list of the current approaches indicating their advantages and drawbacks is provided in the supplementary material, Table 1. Assuming the soft module is prepared with thin-film technology and polymer carrier, connection may be prepared with (1) zero-insertion force (ZIF) connectors,^14^ where the flexible carrier is clipped inside a plastic housing and connected to hard electrical terminals with small levers; (2) anisotropic conductive adhesives in the form of paste or film (ACP, ACF, respectively), applied uniformly over the whole connection area to connect a thick-film flexible printed circuit board (fPCB) to the thin film by alignment of conductive particles embedded in a polymeric matrix upon application of heat and pressure;^12,15–17^ (3) wire bonding from the thin film device to a larger thick PCB;^18^ (4) flip chip or (5) surface mounted device (SMD) assembly; (6) soldering of individual wires to the metal thin film;^19^ or (7) using liquid metals.^20^ Although these methods have proved useful in wearable and implantable prototypes, they rarely meet all the requirements of longevity, durability under mechanical cycling, and high channel counts, and thus would not offer a reliable solution to long-term applications.

Here, we present a versatile connection method, named “FlexComb,” to reliably integrate a structured fPCB with silicone-based soft transducing modules. FlexComb serves both as the electrical interconnection and the compliant interconnect cable. At the interconnection area, the fPCB is machined into fingers, each bearing a contact pad. Similarly, in the silicone encapsulation layer of the soft device, via openings (“wells”) to connection pads are machined with a pattern matching the FlexComb fingers, and filled with conductive paste. At the system assembly stage, the FlexComb fingers are placed and locked into the soft device wells. As each finger sits into its corresponding well, short circuits are avoided and the FlexComb self-aligns to the soft device. Finally, the interconnection area is encapsulated with silicone sealant. This enables low-profile (< 1 mm thick), high-channel count interconnections (we here demonstrate up to 60 channels, but significantly larger numbers are possible), with a connection pitch down to 0.35 mm, a resolution representing significant miniaturization for soft circuits, where the smallest pitch reported is, to the best of our knowledge, 0.2 mm with stretchable ACF.^16^ We implement the FlexComb technique to connect soft neural interfaces to fPCBs with small form-factor. fPCB technology offers higher resolution and lower electrical resistance interconnects compared to current soft interconnect technologies; hence, the hybrid integration reduces the overall footprint of the system and electrical DC load of the interconnects. At the distal end of the FlexComb, a standard (rigid) connector can be mounted or easily integrated with other electronic components using standard fPCB assembly.

We review the assembly process to integrate FlexComb with thin-film soft electronic processing and characterize the connection electrically and mechanically using customized test structures. We then demonstrate soft neural interfaces interfaced with various FlexComb designs and configurations. Finally, we report on the performance of the FlexComb technology over a pilot 6-months chronic implantation study of a surface brain electrode in a minipig model.

## II. RESULTS

### A. Assembly process

**FIG. 1.**
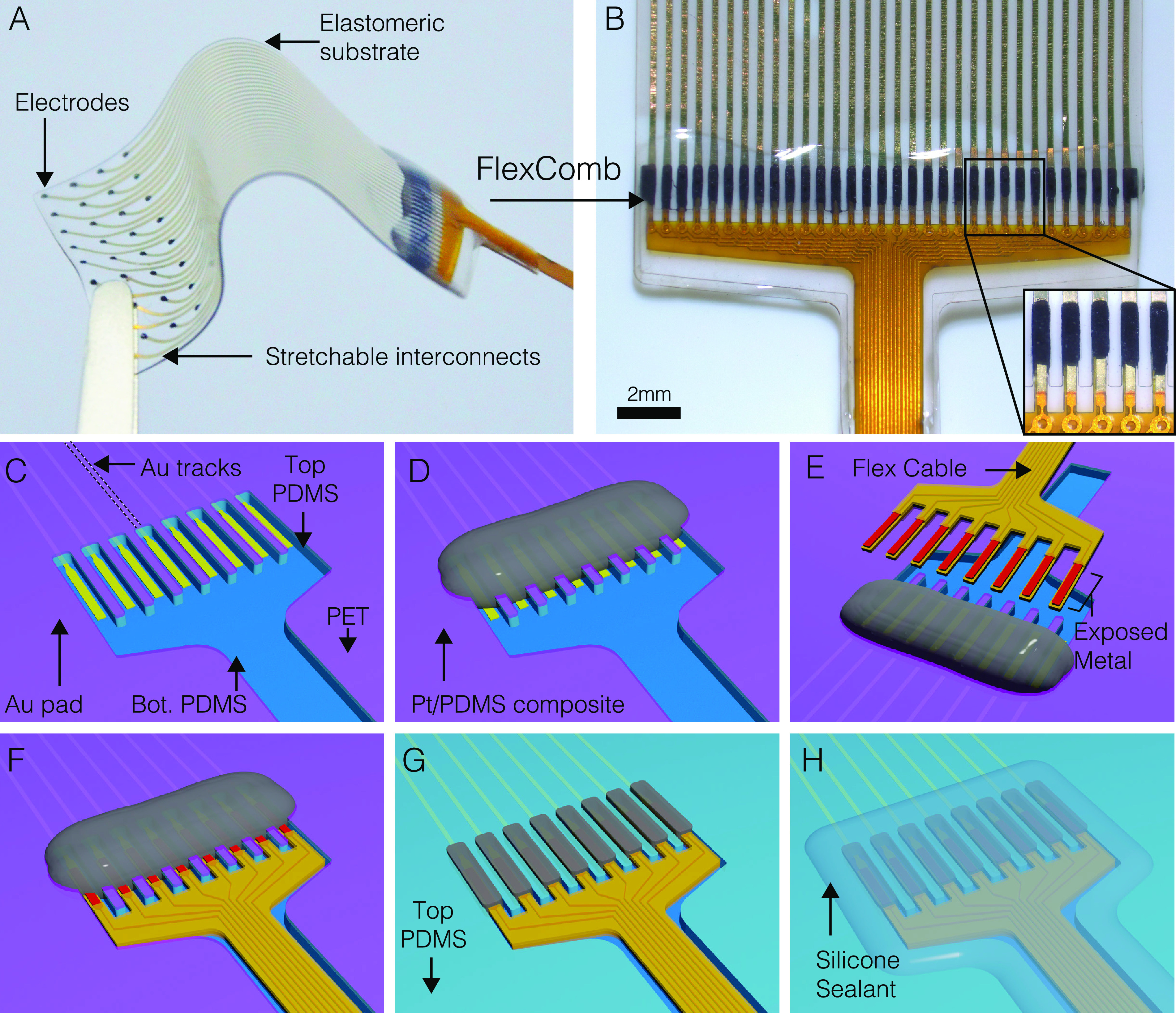
Assembly with FlexComb interconnections. (a) Photograph of a PDMS-based electrode array connected with FlexComb interconnections. (b) Microscope image of the connection area between FlexComb and the PDMS device. Inset: magnified portion of the finger area of the FlexComb. (c)–(h) Illustrated assembly process: (c) post-wafer fabrication of the electrode array on PDMS. (d) Application of the conductive Pt mesoparticles/PDMS composite serving as conductive adhesive over the interconnection area. (e) Alignment and placement of the FlexComb into the PDMS wells. The conductive paste is represented in transparency. (f) Assembly after the deposition of the FlexComb onto the wafer. (g) Removal of the PET stencil mask to pattern the conductive adhesive and isolate separate connections. (h) Dispensing of silicone sealant to mechanically fix the FlexComb onto the PDMS implant and isolate it electromechanically from the external environment. The conductive paste is represented in transparence.

Figure 1 presents the FlexComb connection scheme, applied to a soft module fabricated using the silicone-on-silicon (SoS) process previously presented by our group.^21,22^ The soft module carries stretchable thin-film gold (Au) tracks that require connection to external electronics such as an implantable pulse generator or a neural signal amplifier [Fig. 1(a)]. Each “finger” of the FlexComb connects a soft thin-film track to an electrical track of the fPCB through a conductive paste [Fig. 1(b)]. A silicone sealant completes the hybrid system. The FlexComb provides a relatively thin connector (<1 mm thick) with submillimeter contact pitch. This eliminates the need to fan-out the contact pads on the soft device thereby exacerbating the footprint (width) of the soft module.

The assembly process is presented in Figs. 1(c)–1(h) and further detailed in Figs. 1 and 2 of the supplementary material. The FlexComb is a standard two-layer flexible PCB of overall thickness of 100 *μ*m comprising 18 *μ*m thick copper metallic interconnects. Although copper metallization is not suitable as an implanted material due to corrosion and toxicity considerations, for this proof-of-concept, this material system is deemed satisfactory as the failure time of the insulation protecting the copper interconnects from exposure to ionic/biological media is far larger than any failure that would occur at the interface with the soft device. In Sec. III, we elaborate on possible future work replacing this metal system with biologically safer alternatives. In our example [Fig. 1(c)], the soft module is a PDMS membrane carrying thin-film Au tracks thermally evaporated and patterned through a shadow mask. Next, the membrane is aligned and bonded to a bilayer of a PDMS membrane—polyethylene therephtalate (PET) foil pre-patterned with a laser-machined comb defining the electrical pads. The PET foil is used as a stencil mask. A conductive paste is prepared as a soft composite of platinum (Pt) mesoparticles dispersed in a PDMS matrix^9,23^ and patterned through the stencil [Fig. 1(d)]; alternative soft electrically conductive adhesive may also be used. Here, we use standard uncrosslinked silver paste that can be used in flexible circuits and wearable applications, for example. Next, the FlexComb is aligned to and placed on the soft module, so that the fingers are laid in the PDMS comb and pressed into the conductive paste [Figs. 1(e) and 1(f)]. The stencil PET mask on the top surface is then peeled-off to remove the excess conductive paste and isolate the individual channels [Fig. 1(g)]. Finally, a silicone sealant (silicone RTV) is used to secure the connector mechanically to the PDMS substrate [Fig. 1(h)]. The packaged device is then released from the carrier wafer.

### B. Contact characterization

We assess the contact resistance of the FlexComb technology as well as its change upon mechanical loading. We systematically compare FlexComb samples to standard connector technologies, i.e., a commercial silver paste and surface mounted zero insertion force (ZIF) connectors.

**FIG. 2.**
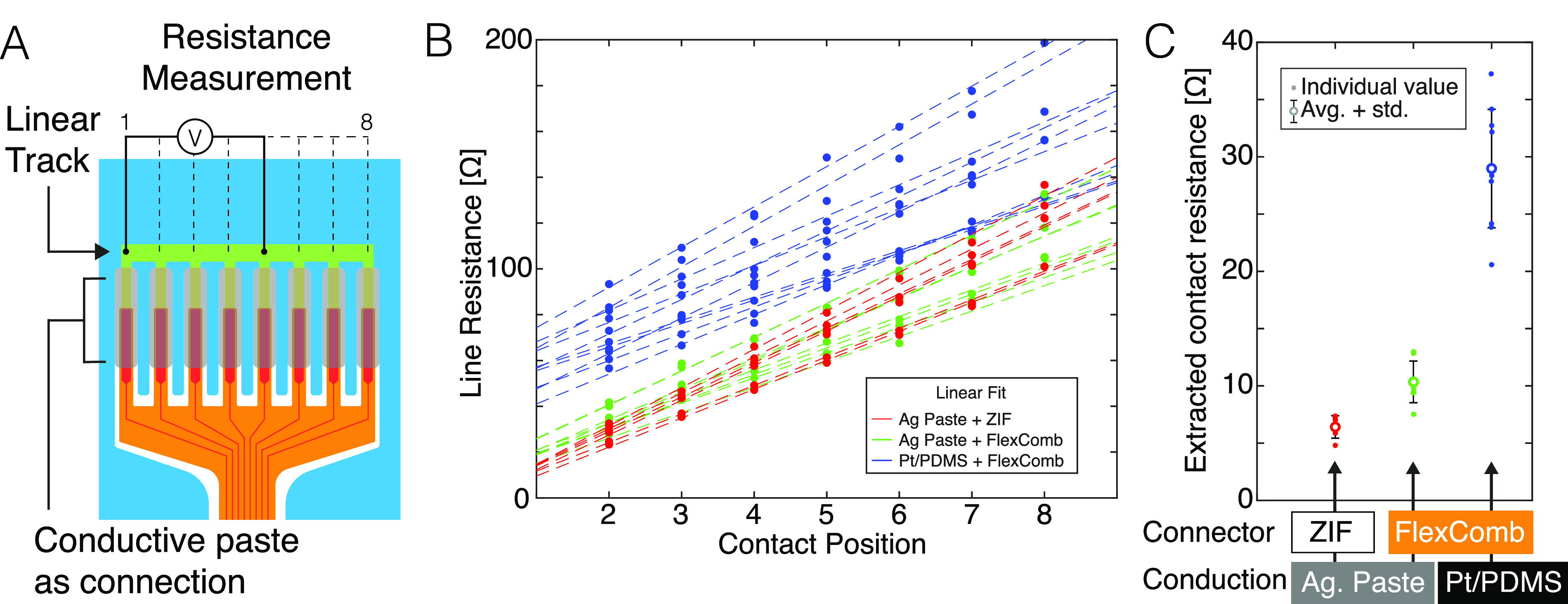
Electrical characterization of FlexComb connections. (a) Diagram of the contact resistance test device. Electrical resistance is measured between each terminal on the distal end of the FlexComb (see supplementary material, Fig. 3). (b) Electrical measurement of the electrical test structure depicted in (a) for three different connection setups: silver paste + ZIF connector (ZIF) (in red), silver paste + FlexComb (in green), Pt-PDMS paste + FlexComb (in blue), respectively. Dotted line: linear regression. (c) Extracted contact resistance for the three connection setups.

We prepare test structures with eight pads (pitch = 0.5 mm, pad width = 0.25 mm) to measure the contact resistance through the transmission line method.^24^ All pads patterned on the soft carrier are shorted with a thin gold bridge [Fig. 2(a)]. The fPCB carries a matching eight-finger comb to be inserted within the Pt-PDMS composite paste. Alternatively, the soft module is connected to a ZIF, or interfaced to the fPCB with silver-epoxy paste, used as comparators.

Two-point electrical resistance is measured through pairs of pads on the distal end of the FlexComb. A reference pad is designated [the first from the left, in the case of Fig. 2(a)], and electrical resistance measurements are taken between the reference pad and all other pads, sequentially. The resistance increases linearly with the pads spacing [Fig. 2(b)]. From the plot, we extract the contact resistance [as the sum of the resistance contributions of the thin Au film to paste, the bulk conductive paste, the FPCB metal film to paste, and the fPCB metal track at “zero length” (pin 0)]. The contact resistance for one pad is then presented in Fig. 2(c).

The resistance of the ZIF connector with the silver paste is low (<10 Ω per contact) as expected due to the low resistivity of the conductive paste and the large pins on the connector. Switching to the FlexComb with the same conductive paste increases slightly the contact resistance (around 10 Ω per contact), which could be explained by the smaller pad area to contact the paste (about 100 *×* 800 *μ*m^2^ compared to millimeter sized in SMT connectors). The average resistance of the FlexComb with the Pt-PDMS is nearly three times higher (around 30 Ω) than with the silver paste, probably due to the metallic particle size and shape in the soft composite. Although this contact resistance is large compared to conventional PCB or connector technology, it is still low compared to stretchable thin-film conductors that have a sheet resistance typically in the 5–10 Ω/sq. range giving rise to track resistances of hundreds of Ohms.^25,26^

**FIG. 3.**
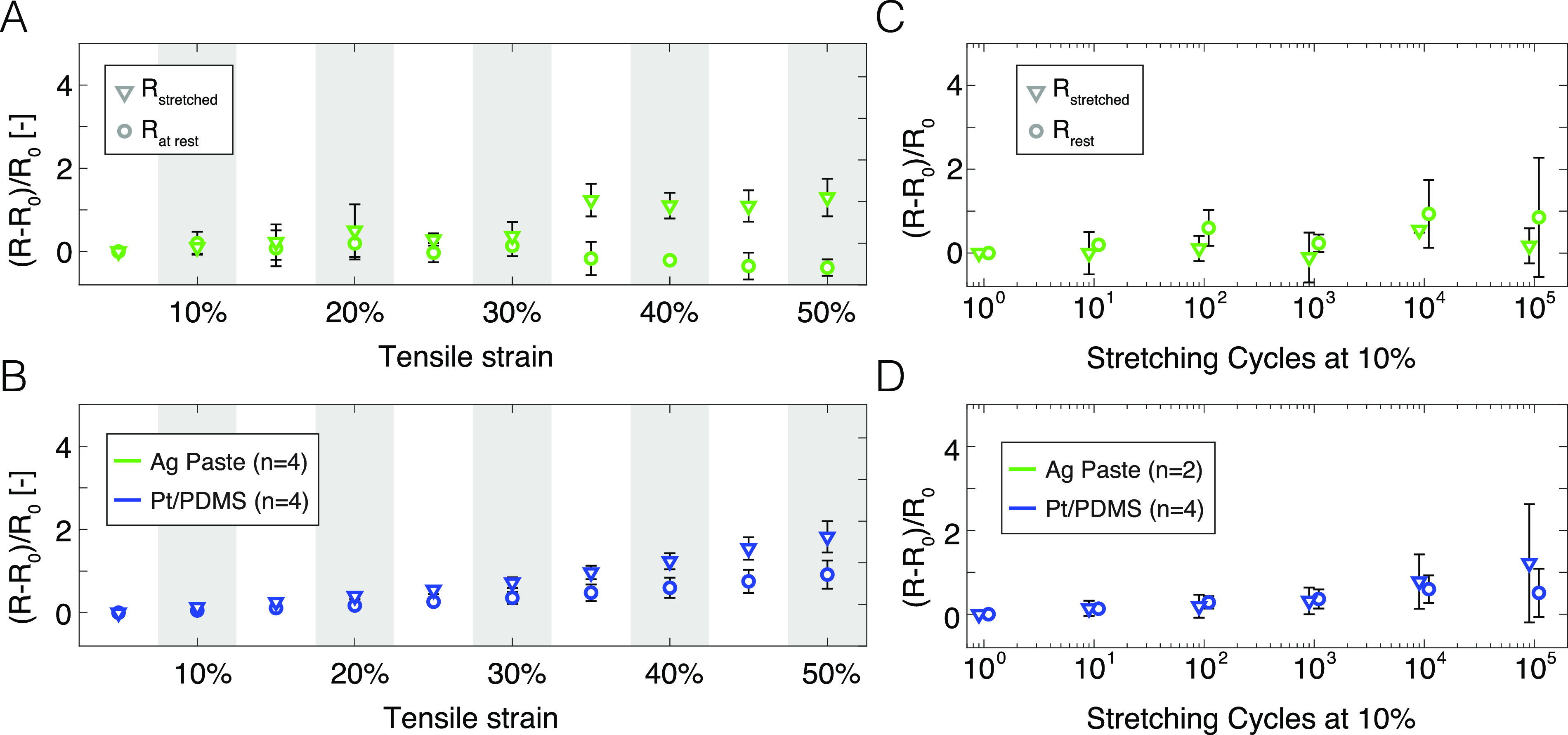
Electromechanical characterization of FlexComb connections. (a) and (b) Relative change of contact resistance when stretched (R, resistance at maximum stretch, diamonds) up to 50% elongation (calculated on the length of the interconnect section) and at rest (R_0_, resistance at rest, circles) after relaxation, for (a) silver paste with FlexComb (green) and (b) Pt-PDMS with FlexComb (blue). (c) and (d) Relative change of contact resistance when stretched (R, resistance at maximum stretch, diamonds) to 10% elongation (calculated on the length of the interconnect section) and at rest (R_0_, resistance at rest, circles) after relaxation, upon cyclic stretching for (c) silver paste with FlexComb (green) and (d) Pt/PDMS with FlexComb (blue).

Next, the electromechanical stability of the assembly is tested under stretching to high strains (>10%) and mechanical cycling shown in Fig. 3. We monitor the electrical resistance of a resistive bridge, as depicted in Figs. 3(a) and 3(b) of the supplementary material, to extract the thin-film track resistance (2-wire and 4-wire configurations) and the contact resistance [supplementary material, Fig. 3(a)]. The clamping and connection scheme is further illustrated in Fig. 3(a) of the supplementary material. The soft tracks are submitted to cyclic stretch up to 50% strain [Figs. 3(a) and 3(b)] and 100 k cycles [Figs. 3(c) and 3(d)]. We compare the performance of the contact resistance of the FlexComb prepared with commercial Ag paste [Figs. 3(a) and 3(c)] and the Pt-PDMS composite [Figs. 3(b) and 3(d)]. The FlexComb sustains reliably the demanding stretching cycles. We observe that, although the relative resistance of the track increases with the applied strain (visible in Fig. 4 of the supplementary material), the change in contact resistance change remains low both at strain and at rest. During cyclic elongation (10% applied strain, 100 k cycles), the contacts remain stable for both tested conductive pastes. The small increase in the contact resistance probably arises from a small resulting stretch of the connection area despite the silicone encapsulation. This effect might be mitigated by increasing the sealant thickness but at the expense of the packaging compactness. Both these electromechanical test results showcase the robustness of the FlexComb solution to reliably connect electrically soft bioelectronic systems.

Next, we probed the strength of the FlexComb during a pull test and found an average 8 N pulling force led to mechanical failure of the system with delamination of the fPCB from the surrounding silicone volume (supplementary material, Fig. 5, n = 8 systems with 8 contacts of 10 mm width, 12 cm length, respectively).

### C. Soaking experiment

Implantation exposes bioelectronic devices to a liquid and ionic surrounding medium, thereby requiring insulation of the conductors and interconnects to avoid degradation, crosstalk, and current leakage between adjacent tracks. We immerse the test samples (FlexComb technology with Ag or Pt-PDMS composite pastes) in phosphate buffered saline solution (PBS 1x) kept at 67 °C for 65 days [supplementary material, Fig. 6(a)]. Each contact pad is connected to one thin Au film track embedded in PDMS. Comparison samples are also connected with individual wires to the device pads using the same silver conductive paste. The DC resistance between each track is measured in saline using a Gigaohmeter at different time points. Over the span of the experiment, the inter-track resistance remains in the GΩ range with stable level, suggesting a reliable encapsulation over the 65 days [supplementary material, Fig. 6(b)]. Comparison to individual wires shows that these are more susceptible to insulation failure mainly due to the serial and manual process required for the wiring.

Furthermore, we observe some corrosion at the Ag paste contacts over time, ruling it out for subsequent *in vivo* use (supplementary material, Fig. 7). Corrosion is not observed on the Pt-PDMS composite over the 65 days.

### D. Implementation to soft transducer systems

**FIG. 4.**
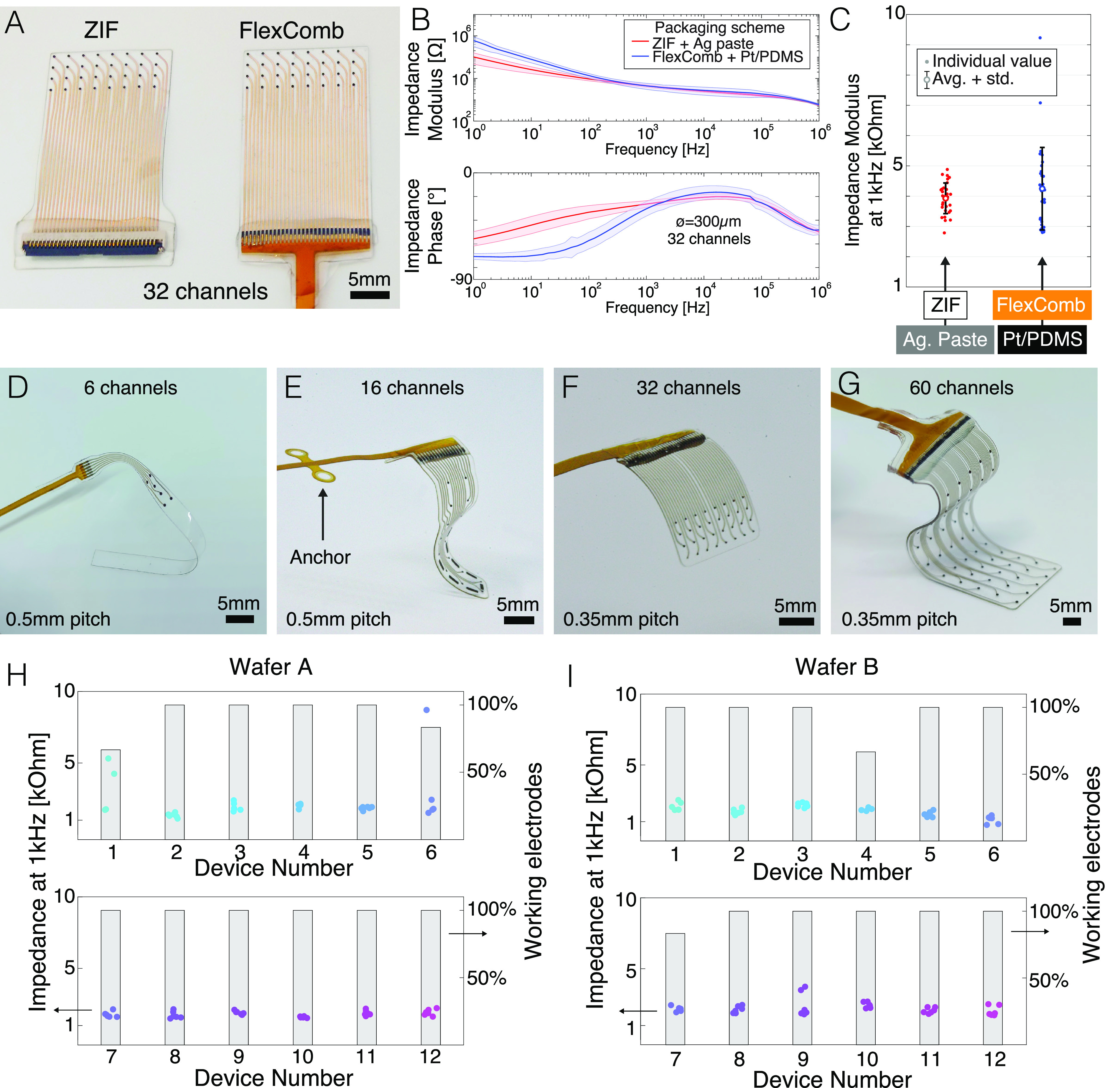
FlexComb packaging for soft electrode systems. (a) Comparison of the surface mounted connector (here, a ZIF connector) (left) and the FlexComb (right) interconnections for 32-channel soft electrode arrays. (b) Electrochemical impedance spectroscopy (top: impedance modulus, bottom: phase) of the electrodes connected with the SMT connector (red) and FlexComb (blue). (c) Impedance modulus values at 1 kHz extracted from (b). (d)–(g) FlexComb configurations for different soft electrode system designs targeting diverse *in vivo* applications. (d) Six-channel rat spinal cord implant with 0.5 mm connector pitch. (e) 16-channel minipig brain implant with 0.5 mm connector pitch and right-angle connection. Anchoring wings for bone screw fixation are shown on the left. (f) 32-channel non-human primate brain implant with 0.35 mm connector pitch and right-angle connection. (g) 60-channel electrode array for the human heart with 0.35 mm connector pitch. (h) and (i) Impedance modulus at 1 kHz (colored dots) and device yield (bar plot) for two consecutive assembled batches, respectively, of the FlexComb with the design presented in (d). An electrode was classified as nonfunctional when the impedance modulus at 1 kHz is >10 kΩ.

Figure 4 displays examples of soft implantable transducers interfaced with the FlexComb technology. 32 electrode arrays are fabricated and connected with two connector technologies: a commercial surface-mounted ZIF connector with silver epoxy paste, and the FlexComb with Pt-PDMS composite paste [Fig. 4(a)]. Each electrode is characterized using electrochemical impedance spectroscopy [Fig. 4(b)]. Impedance spectra show similarity throughout the resistive phase, which indicates negligible difference in the interconnection resistance, while the low-frequency discrepancy is explained by variability in the electrode coating.^27^ Zooming on 1 kHz, all electrodes display comparable impedance values [Fig. 4(c)]. The FlexComb technology, therefore, displays comparable performance to the connector solution we have presented previously.^22^

Figures 4(d)–4(g) illustrate electrode arrays with various footprint and electrode density. Arrays with 6–60 electrodes are connected with flexible inter-contact pitch matching the connection area width and implantation scenario requirements. Our largest device demonstrator comprises 60 channels with a 0.35 mm connection pitch and exhibits functionality of all electrodes with low electrochemical impedance (supplementary material, Fig. 8). The fPCB component of the FlexComb can also be used to implement additional surgical functions, such as anchoring wings [Fig. 4(e)] to secure the implant to surrounding tissue, e.g., bone or muscle.^28^ The fPCB can be designed with a cable exiting the soft module at different angles [e.g., 0° and 90° as shown Fig. 4(e)], as well as serpentine patterns [supplementary material, Fig. 9(a)] to offer further strain relief capability. The FlexComb is compatible with a range of soft electronic technologies such as Kirigami-inspired micro-patterned polyimide/platinum/polyimide layers^26^ [supplementary material, Figs. 9(b) and 9(c)] and viscoelastic electrode arrays (seen in Ref. 5).

Next, we demonstrate the manufacturing reliability of FlexComb connection process using six-electrode soft devices [Fig. 4(d)] patterned and assembled on different wafers processed through different manufacturing runs. The impedance modulus measured at 1 kHz is used as screening benchmark, where channels exhibiting |Z| > 10 kΩ are considered as failed. Figures 4(h)–4(i) display the 1 kHz impedance of all assembled and tested electrodes assembled in two batches. The yield for individual electrodes and complete devices is 95% (138/144 electrodes working) and 83% (20/24 devices fully working), respectively, and low standard deviation of the impedance modulus is reported.

### E. *In vivo* validation

**FIG. 5.**
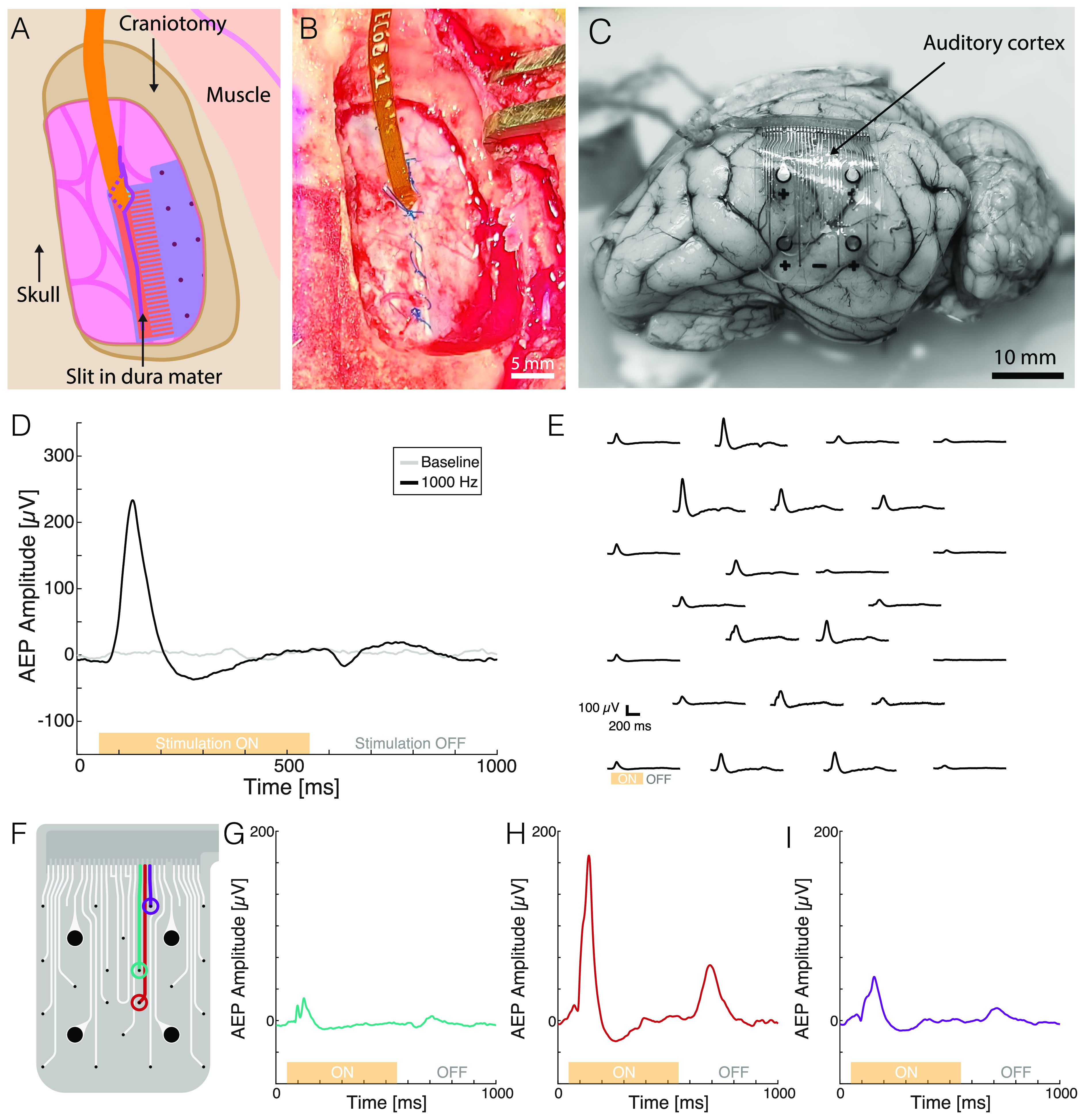
FlexComb as interconnection for *in vivo* neural interfaces. (a) Schematic of the subdural implantation of the neural interface with the FlexComb connector. (b) Photograph of FlexComb implanted subdurally in a minipig model, offering minimal tissue displacement. The dura mater can be sutured around the exiting fPCB cable. (c) Explanted minipig brain after 6-week implantation showing the position of the soft implant and the FlexComb connector. The position of the auditory cortex is highlighted. (d) Representative channel of auditory evoked potential recordings in response to auditory stimuli at 1 kHz compared to baseline after 2 months of implantation. The orange bar represents the timing of the auditory stimuli ON period (500 ms). (e) AEP recording map of the entire electrode array from acoustic stimuli at 1 kHz at 2 months implantation. The orange bar represents the timing of the auditory stimuli ON period. (f) Soft implant layout. Highlighted in color, three channels running in parallel at the connector and interconnect level. (g)–(i) Evoked potential recordings from 1 kHz auditory stimuli at 2 months' implantation from the three channels highlighted in (f). The orange bar represents the timing of the auditory stimuli ON period.

For the validation *in vivo* in a minipig model, an implantable electrocorticography (ECoG) array is designed with soft thin gold film embedded in silicone rubber^22^ and interfaced with a FlexComb connector as presented in Fig. 5. The implant layout is presented in the supplementary material, Figs. 10(a) and 10(b). The 24-channel ECoG array (20 × 20 mm^2^) is implanted subdurally over the auditory cortex.^29^ Thanks to its small form-factor, the fPCB end of the FlexComb can be tunneled through a small slit in the dura mater [Figs. 5(a) and 5(b)], similar to current clinical electrode arrays. After 6 weeks of implantation, the brain tissue shows no macroscopic alteration due to the presence of the soft implant or interconnection system [supplementary material, Fig. 10(c)], demonstrating the compatibility of the FlexComb connector with the surrounding tissue.

The functionality of the soft implanted interface is tested under sedation by measuring auditory evoked potentials (AEPs) in response to acoustic stimuli (500–10 kHz). AEPs are recorded using a wireless amplifier system plugged to the connector on the head of the animal. A representative averaged auditory evoked response to a 1 kHz acoustic tone burst (500 ms ON stimulation—500 ms OFF stimulation) from a single channel is presented in Fig. 5(d) and compared to a baseline with no stimulation. The general shape and parameters of the signal are consistent with minipig AEPs previously described.^30^ At 2 months of implantation, resolutive mapping of the auditory cortex [Fig. 5(e)] could be performed and was comparable to intra-op mapping (supplementary material, Fig. 11). Differences in amplitudes and delay of the ON and OFF responses are visible across the array, showing the ability of the soft implant system to spatially resolve different neural sources from the cortex surface.

A challenge in long-term implantations is the encapsulation stability of the connector system especially at small inter-channel pitches. The hermeticity of the interconnection system in this case can be investigated by analyzing channels running in parallel. Looking at three different channels separated by 220 and 330 *μ*m at the connector and interconnect level, respectively, [Fig. 5(h), green, red, and purple] the recorded brain activity shows very distinct response patterns. This suggests no crosstalk is present; the FlexComb connector is a viable solution at least for medium-term use *in vivo*.

## III. DISCUSSION

FlexComb is a low-profile interconnection technology that answers needs in hybrid integration of soft bioelectronic systems. Contact resistance at the pad level (∼30 Ω) is higher than that of standard electronic assemblies (∼10 mΩ). However, the sheet resistance of soft interconnects is also large (∼1–10 Ω/•^25,31,32^) with typical line resistance > hundreds Ω (for sub-mm wide, cm-long tracks), so the FlexComb contact resistance is comparatively negligible.

Under strain, the FlexComb contact resistance remains relatively stable, exhibiting an increase in a factor < 2 under 50% strain or after 100 k cycles at 10% strain [see Figs. 3(a) and 3(b)]. The higher resistance and electromechanical drift under strain could be improved by engineering further the conductive paste used between the fPCB and the elastic conductors. Although spherical particles (as used here) are ideal for an electrode coating for high-charge injection, they are suboptimal for creating percolating paths in the bulk of the conductive material. The use of asymmetrical or one-dimensional materials, such as nanowires^32^ or flakes,^33^ within the conductive element would reduce the bulk resistance and create a more robust electrical contact under strain.

Furthermore, even though the connection (pad) area is covered by a thicker amount of silicone sealant, the presence of small tensile and shearing forces could still affect the connection. Precise injection molding over the transition area would create a more precise slope between the thin membrane of the device in contact with the target tissue and the flexible cable: a strain-free region on the pad area could be designed to further reduce this effect. Additionally, coating of the flexible cable with silicone along its length could also increase its biointegration by eliminating the sharp edges of the polyimide substrate. Round cross sections could be achieved similar to current medical leads (example shown in supplementary material, Fig. 12).

This work uses off-the-shelf flexible printed circuit boards as connector material, using photo-patternable solder masks and copper as electrical traces, which is unsuitable for long-term implantation and human translation. In fact, copper is toxic in the central nervous system and not stable in saline solutions when exposed.^34^ Furthermore, this encapsulation method has limited water barrier properties, and would certainly degrade over longer implantation periods. Other material systems that can be patterned with similar dimensions (for interconnects and outline geometries, respectively), which offer biocompatibility and long-term stability, should be selected. A notable example is the use of electroplated gold traces embedded in a liquid crystal polymer substrate (LCP) to create the equivalent fPCB geometry to connect to the soft electrode array.^35^ This system has been validated under accelerated ageing conditions and is stable for at least a decade.^36^ In addition, it was tested without adverse events in humans.^37^ FlexComb assembly is compatible with such material technology, and we anticipate application of our technique to biocompatible materials as immediate future work.

Ultimately, the final investigation should be the long-term implantation of a soft electrode array using the FlexComb as a connector and cable in a large animal model as we have presented here in a pilot study. The *in vivo* example presented here serves as a first step to validate the feasibility of our method for chronic use. Test structures could be embedded in the fPCB and implant to follow the evolution of encapsulation properties and contact resistance over time. In addition, the brain section in contact with the FlexComb section should be examined by immunohistology after chronic implantation to investigate the biocompatibility of the connector.

## IV. CONCLUSION

We report on a carefully engineered method to form electrical interconnections between a soft (bio)electronic device and flexible or standard electronic wiring boards. One of the most prominent application spaces for this technology is the field of implantable soft bioelectronic devices. Significant achievements have been published in maturing soft technology for bioelectronic interfaces, yet much less attention has been given to the necessary interconnection technologies. There is, however, consensus in the field that this is one of the most crucial challenges that must be addressed to ensure future long-term translational application of new devices in large animal models and ultimately to humans.^38^ The technological developments presented herein represent promising initial steps toward a scalable manufacturing solution for high channel count implantable interconnections to soft bioelectronic devices.

## V. METHODS

### A. Assembly of the FlexComb connector

The process flow to wire soft implants with the FlexComb connector technique is presented in Figs. 1 and 2 of the supplementary material. First, a wafer with conformable electrode arrays is processed as presented previously.^21,22^ The design of the contact pad opening is matching the design of the fPCB where parallel “wells” are placed next to each other and join for a larger opening that can receive the fingers (in the wells) and cable portion (in the wider area) of the flexible PCB, respectively. The FlexComb is designed on a PCB CAD software (Eagle, Autodesk) and manufactured by a commercial PCB shop (PCBWay, China). The fPCB is comprised of a stack of coverlay/adhesive (25 *μ*m) over a copper/adhesive/polyimide core/adhesive/copper stack (50 *μ*m) with an adhesive/coverlay (25 *μ*m) at the bottom. The copper layers are 18 *μ*m thick, and the overall thickness is around 100 *μ*m as measured on the received product. First, a small drop of silicone sealant (NuSil 4244) is placed at the wider portion of the opening to secure the FlexComb from the bottom. In the well area above the gold contact pads, a conductive paste (silver paste (Epotek) or a mix of Pt nanoparticles (Heraeus) and PDMS (NuSil 4244) is dispensed or screen-printed across all channels. Then, the FlexComb is aligned and placed, so each finger is placed inside a well. Then, additional conductive paste is dispensed or screen-printed over the exposed metal area. The excess conductive paste is removed with a blade and the PET screen-printing layer is removed (to remove the shorting conductive paste). Finally, silicone sealant (NuSil 1540) is poured over the connection area to electrically isolate the contacts and mechanically anchor the fPCB to the silicone substrate. This is replicated for all devices present on the wafer. In the case of the Pt-PDMS conductive paste, the matrix is cured at 55 °C for 4 h after overnight at room temperature. The device outlines are then cut with a femtosecond laser (Optek, Belgium) and released in de-ionized water.

### B. Electrical measurement

The two-wire (2 W) and four-wire (4 W) measurements are carried out with a source meter unit (Keithley 2100) using 5 V as input.

### C. Mechanical test

For uniaxial tensile tests, customized mechanical clamps are fixed on a tensile test machine (MTS Criterion C41 with a 100 N load cell). The device is placed in the clamps and stretched at 0.1 mm/s. The electrical measurements are performed at the maximum elongation and at rest as described above. For the cyclic stretching experiment, the sample is fixed in a customized mechanical cycling machine and stretched to 10% elongation at 1 Hz.

### D. Electrochemical impedance

The impedance of the electrode is measured as a function of frequency in a three-electrode setup in phosphate buffered saline (PBS). The electrode under test is the working electrode (WE), a platinum wire is the counter electrode (CE), and an Ag/AgCl electrode (Metrohm) is the reference electrode (RE). With a potentiostat (Gamry Instruments 600), a sinusoidal voltage signal with a specified amplitude (VAC = 100 mV) is applied between the WE and CE. The voltage of the WE vs RE and the current flowing through the WE-CE circuit are measured, and their ratio and phase delay calculated. The frequency of the excitation signal is swept from 1 MHz to 1 Hz with 3 points per decade.

### E. Leakage current measurement

The insulation resistance is measured with a Gigaohmmeter (Sefelec) with the sample immersed in the aging vial filled with saline (PBS 1x). A 300 V potential is applied for 15 s to stabilize the reading and then the resistance is extracted.

### F. *In vivo* experiment

The following procedure has been presented in detail in Ref. 29. In short, a Göttingen minipig of 8.5 kg (3 months old) was anesthetized with a pre-medication mix of Haldol (0.1 mg/kg), midazolam (0.75 mg/kg), and atropine (0.25 *μ*g/kg) and induced with 5% sevoflurane. After intubation, an intravenous line is placed on the ear for continuous administration of 10 ml/h propofol at 2 wt. % and along a bolus of 2 *μ*g/kg fentanyl during the heavy surgical procedures. The surgery was performed as follows: a large frontal to posterior incision is made over the midline. The muscle is retracted, and the periosteum is removed using an elevator. The midline and bregma are identified. The craniotomy is planned 2 mm laterally from the midline to 20 and −10 to +20 mm anterior to frontal. The skull is drilled with a neurosurgery drill (Bbraun Elan 4 using a 2.5 mm neuro cutter drill bit) until reaching the dura. Then, the bone flap is broken free using a spatula. The dura mater is incised with a microscalpel (stab scalpel knife, Fine Science Tools) in a slit. The soft implant is inserted using flat tweezers until the connector sits under the dura mater slit. The dura mater is then sutured around the connector and cable area (Ethicon, Mersilk 5–0). The bone flap is then placed back and secured using titanium mesh and screws (Medtronic Ti Mesh system). The connector headstage is then secured using titanium screws in the bone. The skin is sutured back around the transdermal port and cleaned. Post-operative care consisted in a buprenorphine patch (25 mg/h) applied for 24 h. A dose of cephalexin at 75 mg was given daily with food for prevention of infection.

### G. Electrophysiology

The brain signals were acquired by connecting the FPCB on the implant through a surface mounted connector (Omnetics) to a wireless headstage amplifier system (Multichannel Systems Wireless W2100). A ground wire is inserted epidurally posterior to the dura mater slit. The acoustic stimuli is delivered with tone bursts at different frequencies (500–10 kHz) with 500 ms ON and 500 ms OFF periods through in-ear speakers (Etymotic). The acquisition is performed at a sampling frequency of 2 kHz. The signal is then filtered with a digital Butterworth bandpass filter of 1–100 Hz and a notch filter at 50 Hz. The recorded signals were averaged over each individual stimulation pulse for a total of 120s (roughly 60 epochs per condition).

## SUPPLEMENTARY MATERIAL

See the supplementary material for more details on the fabrication of the FlexComb connection, the electromechanical sample geometry and measurement setup, mechanical results, soaking results, FlexComb geometry variations, and complementary *in vivo* results.

## Data Availability

The data that support the findings of this study are available within the article and its supplementary material.
